# Supplementary material for: Ultra-Wide-Field Fluorescein Angiography Assessment of Non-Perfusion in Patients with Diabetic Retinopathy Treated with Anti-Vascular Endothelial Growth Factor Therapy
Source: J Clin Med. 2023 Feb 8;12(4):1365. doi: 10.3390/jcm12041365 (PMC9963628; doi:10.3390/jcm12041365)
Supplement: Supplementary file 1 [file jcm-12-01365-s001.zip › jcm-2158623-supplementary.pdf]

Table S1. Patients' baseline demographics of analysis population and population not included in the analysis.

Variable	Analysis population N=20	Population not included N=28
Mean age, years, (± SD)	63 ± 11	68 ± 9
Male gender, n (%)	13 (65%)	18 (64%)
Right eye, n (%)	13 (65%)	14 (50%)
Median BCVA, ETDRS letters [IQR]	70 [59; 77]	68 [61;75]
Median CMT on OCT, μm, [IQR]	324 [287; 471]	357 [310;445]
Type 1 diabetes, n (%)	0 (0%)	2 (7%)
Median Hba1c, % [IQR]	8 [7; 8]	8 [7;9]
Patients treated with insulin, n (%)	11 (55%)	18 (64%)
Macroangiopathy, n (%)	4 (20%)	5 (18%)
DR stage, n (%)		-
Moderate	7 (35%)	12 (43%)
Severe	13 (65%)	16 (57%)
Previous treatment for DME, n (%)		-
none	20 (100%)	25 (89%)
anti-VEGF	0 (0%)	2 (7%)
corticosteroids	0 (0%)	0 (0%)
laser	0 (0%)	1 (4%)
Previous PRP, n (%)	0 (0%)	0 (0%)
Lens status: phakic, n (%)	12 (60%)	16 (46%)
HBP, n (%)	7 (37%)	14 (50%)
Renal status, n (%)		-
No renal failure	16 (84%)	22 (79%)
Microalbuminuria	2 (11%)	1 (4%)
Renal failure	1 (5%)	5 (18%)
Dyslipidemia, n (%)	6 (33%)	14 (50%)
Sleep apnea, n (%)	1 (6%)	1 (4%)
OHT or glaucoma, n (%)	3 (15%)	2 (7%)
